# Accurate Non-Disturbance Population Survey Method of Nesting Colonies in the Reedbed with Georeferenced Aerial Imagery

**DOI:** 10.3390/s20092601

**Published:** 2020-05-02

**Authors:** Gábor Bakó, Zsolt Molnár, Zsófia Szilágyi, Csaba Biró, Edina Morvai, Örs Ábrám, András Molnár

**Affiliations:** 1 Interspect Ltd., II. Rákóczi Ferenc út 42, H-2314 Halásztelek, Hungary; bakogabor@interspect.hu (G.B.); molnarzsolt@interspect.hu (Z.M.); szilagyizsofia@hotmail.com (Z.S.); 2 Kiskunság National Park Directorate, Liszt F. u. 19, H-6000 Kecskemét, Hungary; birocs@knp.hu (C.B.); edi@kolon-to.com (E.M.); 3 Moving Sand Nature Conservation Association, Matyó dűlő 46, H-6070 Izsák, Hungary; orsabram@gmail.com; 4 John von Neumann Faculty of Informatics, Óbuda University, Bécsi út 96/b., H-1034 Budapest, Hungary

**Keywords:** aerial survey, heron colony mapping, remote sensing, population counting

## Abstract

High altitude aerial surveys have the potential to improve disturbance-free data collection in wildlife research, but previously, bird species were not recognizable in high-altitude orthophotos. This method of aerial surveying is effective and can be repeated frequently due to its low cost; it also has the additional advantage of being able to monitor the status of protected areas. In the case of waterbirds, due to the low vegetation coverage, aerial remote sensing is an exceptionally effective technique for surveying populations and detecting nests. Aerial surveys made at low altitudes can cause serious stress for birds. The method we developed and employed is unlikely to be detected by either ground-based or nesting birds but is far more reliable compared to the low-resolution imaging methods and to the evaluation of non-georeferenced photo series. The modern sensors and photogrammetric procedures enable the use of the present method worldwide; furthermore, the large-scale ortho image-derived information has become obtainable more frequently. Direct georeferencing makes the field geodetic survey unnecessary. Orthophotos with a 0.7 cm spatial resolution allow us to reliably identify even the individuals of smaller species, and by the use of oblique images, they can be tracked from two or four different directions.

## 1. Introduction

From the perspective of wildlife conservation, it is very important to develop such monitoring systems that are able to reveal the changes in the environment. Wetland systems are vulnerable to changes in the quantity and quality of their water supply, and specific restoration and management plans will require examinations by habitats [1].

The success of reliable and frequently sampled monitoring procedures are based on working with indicators that can be credibly tracked in space and time [2,3,4]. In the case of remote sensing methods, amongst the examples are examining the top foliage level of forest reserves, mapping the patch dynamics of grasslands, continuously monitoring the propagation vectors of invasive plant species, and surveying the waterbird colonies by aerial status surveys during the breeding seasons. Aerial mapping not only assists in determining bird populations but can also give useful information in relation to the surrounding vegetation and the nesting site habitat [5]. A close relationship can be found between the vegetation and the nesting habits [6].

**Figure 1:**
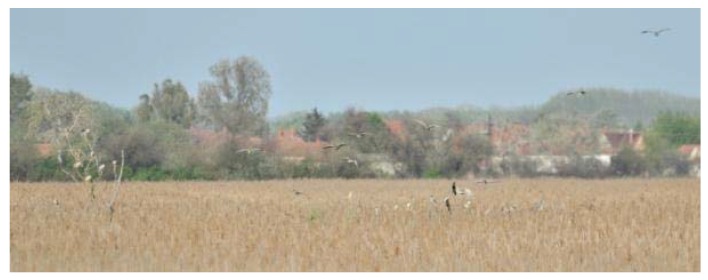
In the case of field counting, the birds will detect the approaching ornithologists from a long distance. Counting the individuals that are leaving or arriving at the site does not provide exact information about the size of the populations (capture by Örs Ábrám).

In the field, we can deduce the number of nesting pairs at the heron colonies at dusk or at dawn, when they are flying above several square kilometers of dense reeds or areas with muddy soil. On an impenetrable terrain, when counting from the ground, in smaller sub-areas after counting the occupied nests and determining the proportion of nesting species, the results can be projected to the entire area of the colony. We can count the nests within the 5 m bands marked on large colonies and summarize them [7]. In these cases, it is not possible to count the individuals due to disturbance. In binocular counting (Figure 1), the number of flying in and out birds is divided by four because they are likely to double count. However, these methods do not accurately return the number of individuals, or the statistics between years may be distorted.

During the latter half of the 20th century, aerial-based surveys were being utilized to observe and study breeding sites, but due to the limitations of the camera equipment and particularly the resolution, it was necessary to fly close to the nests, causing disruption.

Due to the physical approach, these aerial surveys have also disturbed the colonies. Therefore, it is worth examining how to perform high-spatial-resolution data acquisition from higher altitudes using airplanes and UAVs (Unmanned Aerial Vehicles).

During the period of 2006–2014, we developed a method with a large spatial resolution that allows us to create a contiguous orthophoto map with subcentimeter spatial resolution from a high altitude (450–800 m) of a few km^2^-large areas [8]. Since that time, we have successfully applied this method for surveying several Central European breeding sites. We present the survey’s spatial resolution dependence through a survey of Lake Kolon that was made in 2018. Based on our experience of the past several years, we would like to draw attention to the justification of disturbance-free surveying.

Therefore, we would like to introduce a long-term monitoring procedure, undertaking at least three monitoring surveys a year for each breeding site, with the aim of introducing a completely disturbance-free procedure.

The area of Lake Kolon at Kiskunság National Park is the largest natural wetland of Kiskunság. The 1200 ha large reedbed lake is bordered by sand dunes, meadows, and swamp woodlands, which have high natural values. The area is also part of the Ramsar sites, which are protected wetlands. Colony species that are breeding there today consist of Great White Egret (*Egretta alba*), Little Egret (*Egretta garzetta*), Squacco Heron (*Ardeola ralloides*), Black-crowned Night Heron (*Nycticorax nycticorax*), Grey Heron (*Ardea cinerea*), Eurasian Spoonbill (*Platalea leucorodia*), and Pygmy Cormorant (Phalacrocorax pygmeus). Sparsely breeding species consist of Purple Heron (Ardea purpurea), Common Little Bittern (*Ixobrychus minutus*), and Eurasian Bittern (*Botaurus stellaris*). 

Unforeseen weather at the time of migration add factors, which can cause uncertainties, changing the population size [9], but at the same time, we can gain information on the quality of the habitat as well by examining the resettlement or the changes of colonies regarding their structure and size. The state of each population is an indicator of the environmental status of the area [10], and the splitting of the habitat may lead to the suppression of the species [11]. Similar observations point out that not only the counting of the nests but also the counting of the individuals should be performed at the breeding sites.

## 2. Method

**Figure 2:**
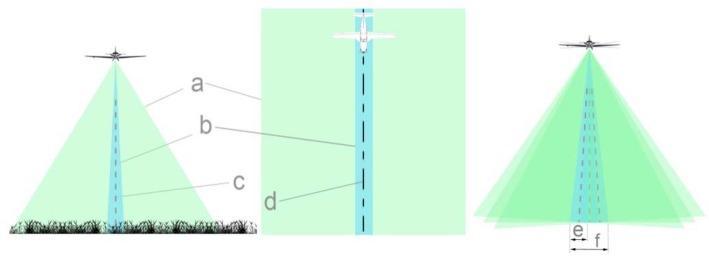
The preliminary low-resolution aerial survey’s 54.43° horizontal angle of view (**a**) and swath width ratio, the high spatial resolution aerial survey’s vertical axis aerial camera’s 4.1° horizontal angle of view (**b**) and swath width ratio, the camera axis (**c**), and the swath centerline (**d**). It’s important to prevent rotation of the rolling shaft, especially in the case of the high-resolution survey, because of the displacement distance (**f**) and swath width (**e**) ratio.

The aerial survey begins with a preliminary low-resolution aerial survey, which we use to monitor the changes in the size of the breeding colonies and whether nests have appeared in new areas. After the localization of the nesting sites, we plan the flight of the high-resolution survey. For the 36 × 24 mm sensor-sized high-speed aerial camera system, we use different lenses from 300 to 650 mm focal length. During a survey at 450–800 m above surface level, besides maintaining the aircraft flight lines, it is highly important to compensate for the roll rotation and to ensure the great overlap of the recordings (Figure 2).

For example, a lens with a focal length of 500 mm achieves a swath width of 43.1 m from an altitude of 600 m.

At this altitude, when the aircraft rolls at 1 degree, the swath slides 10.47 m away.

We conducted a study and found that 13.35 percent of the shots showed a more than a 5-degree deviation from the vertical camera axis in case of roll on the longitudinal axis during pilot-driven flights with the fixed camera. This was 205 images out of 1535 shots.

Due to the narrow-angle of view, therefore, the use of Autonomous Driving Systems [12,13,14] is required. For example, the SkyView HDX autopilot roll hold mode holds the current aircraft roll angle. Autopilot controls the roll hold, and ground speed cruise control uses externally mounted DGPS (Differential Global Positioning System) to provide superior accuracy. The roll target is the current roll angle of the aircraft at the time roll hold is activated, but the roll target can be adjusted with control wheel steering.

It is essential that the SV-ADAHRS (high accuracy air data, attitude, and heading reference system attitude sensor) is installed correctly, calibrated, and operating well at all altitudes.

AHRS (Attitude and Heading Reference System) can be combined with air data computers to form an air data, attitude, and heading reference system (ADAHRS), which provide additional information, such as airspeed, altitude, and outside air temperature. ADAHRS installation location should be a rigid surface within 12 feet longitudinally (front-to-back) and 6 feet laterally (side-to-side) of the aircraft’s center-of-gravity. Typically, the accuracy expected is within ±0.5 degrees. ADAHRS in case of roll accuracy is under ±0.02 degrees between −20 to 70 °C in linear flight. State sensor technology delivers roll hold accuracy to within 2 degrees in all experienced weather conditions.

Recording speeds with redout speed (minimum of four frames per second) and 4.87 microns CMOS (Complementary Metal–Oxide–Semiconductor) allow for extremely high spatial resolution images (Table 1 and Table 2). Given a target feature recognizable sign’s size and ground sample distance based on mission objectives, the flight height and velocity should be calculated to ensure motion blur is kept to a minimum [15]. The shutter speed is fast enough to minimize motion blur in the case of ISO 320, which results in a low noise level. The swath width (Table 3), on the other hand, is narrow and justifies the autonomous autopilot flight described above. In the future, we can expect even faster readout speeds [16].

The system records the position and tilt of the camera during the exposure, which means the x, y, and z location of the camera’s projection center point and the yaw, pitch, and roll of the camera axis. The orientation of the camera is given by omega (the rotation about the *x*-axis), phi (the rotation about the *y*-axis), and kappa (the rotation about the *z*-axis). The coordinates, the altitude, and the omega, phi, and kappa angles are collectively referred to as the six parameters of external orientation [17,18,19,20]. Since we cannot measure GCPs (ground control points) in the reeds, the direct georeferencing is essential for the orthophotos. The images and the measured orientation data also allow the automatic determination of camera interior orientation parameters, and it is an automated checking of the optical system [21].

### 2.1. Geometric Accuracy of the Surveys

When designing flight paths and photography base points, the accuracy of the acquisition grid shape and the acquisition grid geometry parameters (pitches, image rates, camera framing, flight heights) and 3D photogrammetric surface reconstruction accuracy must be considered because the best combination of grid shapes and acquisition grid geometric parameters determine the desired accuracy for the required GSD (Ground Sample Distance) [22].

Geometric verification was performed on a land-based test field in order to determine the geometric properties of the method (Table 4). This easily accessible test site is necessary because accuracy assessment cannot be performed in the reedbed. It should be noted that in the case of reedbed, photo align is more difficult to perform by SfM (Structure from Motion) photogrammetric bundle adjustment than in the grassland used as the test area.

### 2.2. Evaluation

Orthophotos and the oblique camera-axis images are evaluated manually in GIS software in triplicate by different analysts. The final result is a composite of the evaluation. Individuals and nests are interpreted in a point-type vector-graphic map layer.

Due to the geometrical accuracy of high-resolution technology, no motionless individual can disappear when the images are overlapped. However, orthophotos are also analyzed without mosaicking as individuals can be seen from multiple angles, and the movement of flying individuals can also be determined. Moving individuals, especially flying ones, require manual inspection at the linking points of the images. The high flight speed and the rapid return between the flight rows are not important during the counting of nests but are important throughout the counting of colonies and those individuals that are surrounding them.

The reliability of the results from aerial surveys can depend on the experience and training of the observers, as well as the variation in the detectability of different species [23,24,25].

### 2.3. Identification of the Typical Heron Species of Lake Kolon

The feet of the Little Egret is yellow, which allows us to distinguish it from the Great White Egret during its flight, whose legs are dark brown or black. The pastern of the Little Egret is black, while the Great White Egret’s pastern is yellow—these can’t be seen from a top view. The beak of the Great White Egret is yellow beside the breeding period when it is black, but its facial skin is green at all times. By contrast, the Little Egret’s beak is always black.

The Great White Egret builds its nest in areas where the reeds are densest, forming sites with its companion species. Its nests are made of dry reeds; for this purpose, the Great White Egret uses the surrounding reeds. The Great White Egret builds its nest densely, but only the middle of the nest is more robust, where it is lined with dry reeds. Occasionally, it puts the nest on willow bushes, which can be found in swamps; in this case, the nest is smaller, and it consists of branches.

The Little Egret breeds in mixed heron colonies. Typically, it nests with Squacco Herons, Black-crowned Night Herons, and Glossy Ibises (*Plegadis falcinellus*). Its nest is made of willow branches and can be found in willows or bushes. Its nest is small and very loose compared to the bird’s size. Later, during the breeding and nestling rearing period, the Little Egret lines the nest with fresh, leafy branches.

Egrets breed once per year. The Great White Egret lays 3–4 eggs, while the Little Egret lays 3–6 eggs. If the adult bird is not staying at the nest, it is almost impossible to distinguish the nestlings of the two species on an orthophoto; furthermore, the Eurasian Spoonbill’s nestlings also have yellowish beaks. The larger Great White Egret nestlings can be identified by their posture and size.

**Figure 3:**
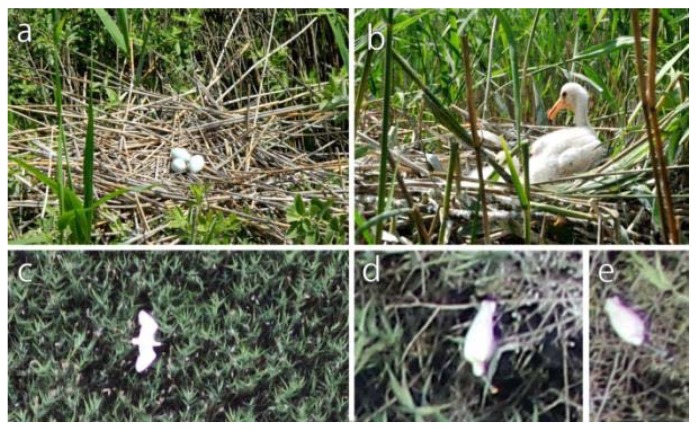
The Eurasian Spoonbill’s (**a**) eggs, (**b**) nestling, and the Eurasian Spoonbill in a (**c**) flying and in (**d**,**e**) sitting position. (capture by Örs Ábrám, Edina Morvai and Gábor Bakó).

If Eurasian Spoonbills are present in large numbers, they construct pure Spoonbill sites, but they occasionally create mixed colonies with other types of herons. The Eurasian Spoonbill builds its nest lower than other heron species. Its large, flat nest is very durable. For this purpose, the Eurasian Spoonbill breaks the surrounding reeds, and then both parents carry reeds and bulrush leaves to the nest. It also uses seaweed, tussocks, and other aquatic plants for the lining. The breeding season of the Eurasian Spoonbill is in May and June. It lays 3–4 rough-grained, pallid, white eggs, which have several red patches and dots. There can be significant differences in size among the nestlings. At the age of 4–5 weeks, they are already walking around the nest, but they only fly out of the heron colony at the age of 7–8 weeks. In the aerial photos, the larger nestlings look very similar to the great white heron nestlings, which have yellow beaks, because their size and color are similar (Figure 3).

The widening part of the Eurasian Spoonbill’s beak is occasionally yellow on the orthophotos, but not in all cases. On top of its back, a yellow spot can often be observed.

The Pygmy Cormorant’s adult and young individuals can hardly be differentiated on the orthophotos. The black color of the individuals in cases when the water is the background makes it more difficult to count the birds.

Adult Grey Herons can easily be confused with Purple Herons, while young individuals may be confused with adult Black-crowned Night Herons in small and medium resolution images. The Grey Heron builds its one diameter large nest from branches, its cup is lined with wool and feather, and it is usually situated in the branches of tall trees. It also breeds in the reeds using aquatic plants and reeds to build its nest.

**Figure 4:**
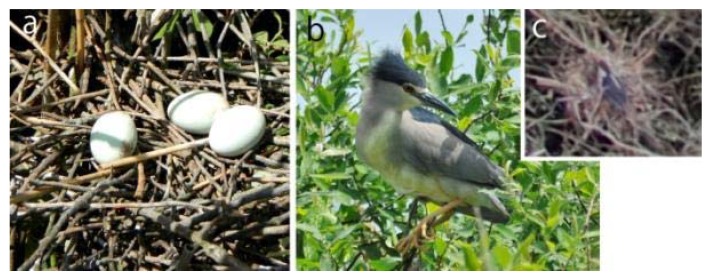
The (**a**) nest of the Black-crowned Night Heron, (**b**) the adult bird, and (**c**) an adult bird, sitting on its nest—details of an orthophoto (captures by Edina Morvai and Gábor Bakó).

The counting of Black-crowned Night Herons (Figure 4) is difficult in bushy areas. They create large colonies and often nest with Little Egrets or Squacco Herons. Its crow’s nest-sized nest, which is situated on the side branches of bushes and trees, are made of dry branches and lined with bulrush or reeds.

**Figure 5:**
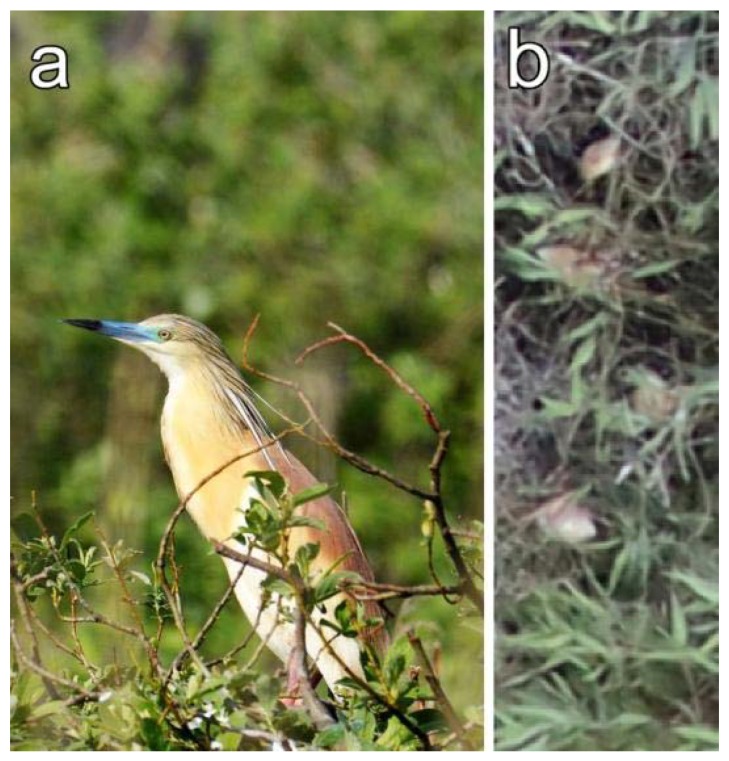
A Squacco Heron captured (**a**) on-site and (**b**) from above (captures by Edina Morvai and Gábor Bakó).

The secure detection of the Squacco Heron is not possible based on aerial photographs, which have a resolution of less than 1 cm (Figure 5).

The Squacco Heron prefers to build its nest in the willows of swamps, floodplains, and gallery forests. It is made of thin branches and is slightly lined. It rarely breeds in reeds if bushes can be found in them. In those cases, it builds its nest from reed fragments. The Squacco Heron nests in colonies, generally mixed with Little Egrets and Black-crowned Night Herons.

Amongst the other bird species that can be found in the area, we could identify the Purple Heron from aerial photography.

## 3. Results

**Figure 6:**
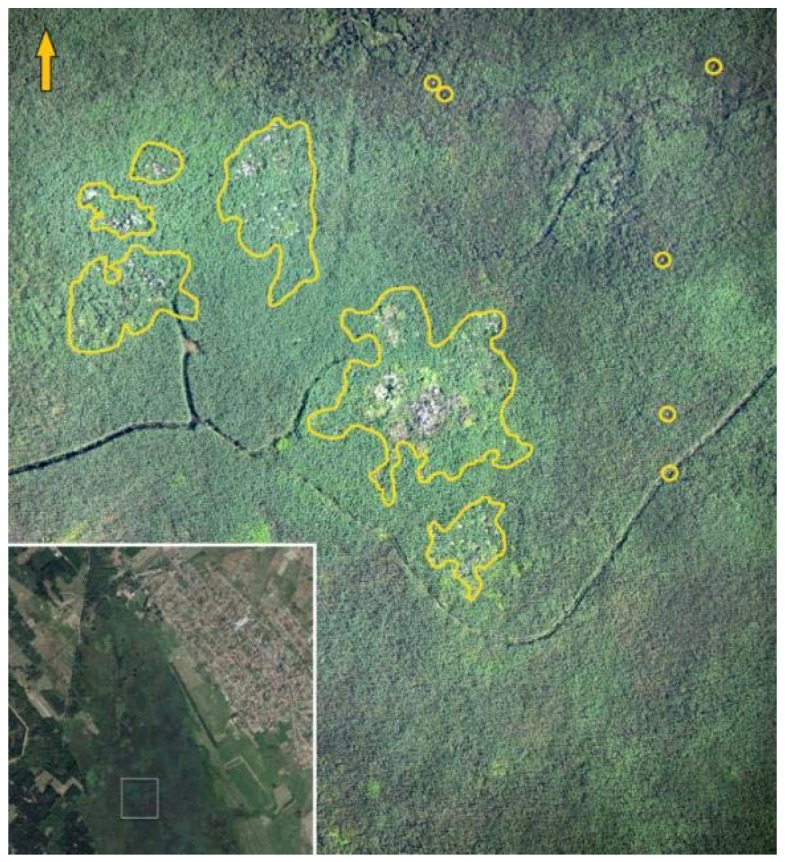
Breeding sites at Lake Kolon. The analysis of Lake Kolon’s heron colonies through an orthophoto mosaic with a medium resolution (7.5 cm).

In the orthophoto mosaics between 7–10 cm GSD (ground sampling distance) range, we identified six major coherent breeding sites at Lake Kolon (Figure 6) in a 55 km^2^ large area. Some isolated nests occurred far from this area.

**Figure 7:**
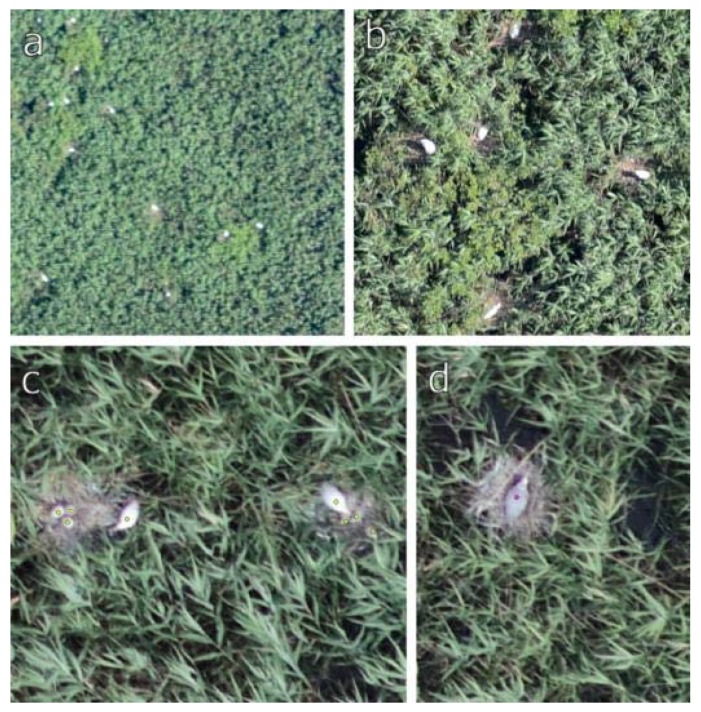
Comparison of the orthophotos with different spatial resolution. (**a**) Great White Egrets in the details of a medium (7.5 cm GSD) and (**b**) a high resolution (1.5 cm spatial resolution) orthophoto mosaic. (**c**) Great White Egrets and (**d**) a Grey Heron in its nest in the details of an orthophoto with 0.7 cm spatial resolution. GSD, ground sampling distance.

The distinction of White Herons from other species was feasible from a 2.5 cm spatial resolution, and heron species could be distinguished at a 0.7 cm large spatial resolution (Figure 7).

**Figure 8:**
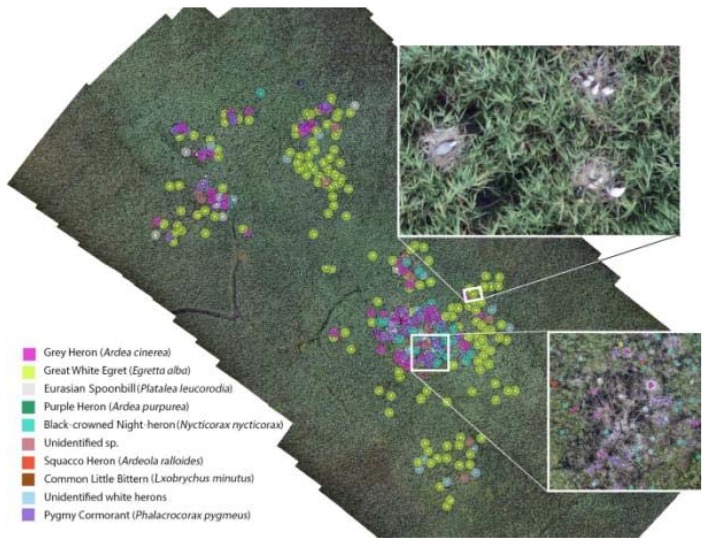
The resulted colony map. An orthophoto mosaic with 0.7 cm spatial resolution with an indication of the detected individuals (points) and nests (full circles).

The 0.7 cm spatial resolution orthoimages of heron colonies at Lake Kolon and the interpretation of the individuals and the nests are shown in Figure 8.

**Figure 9:**
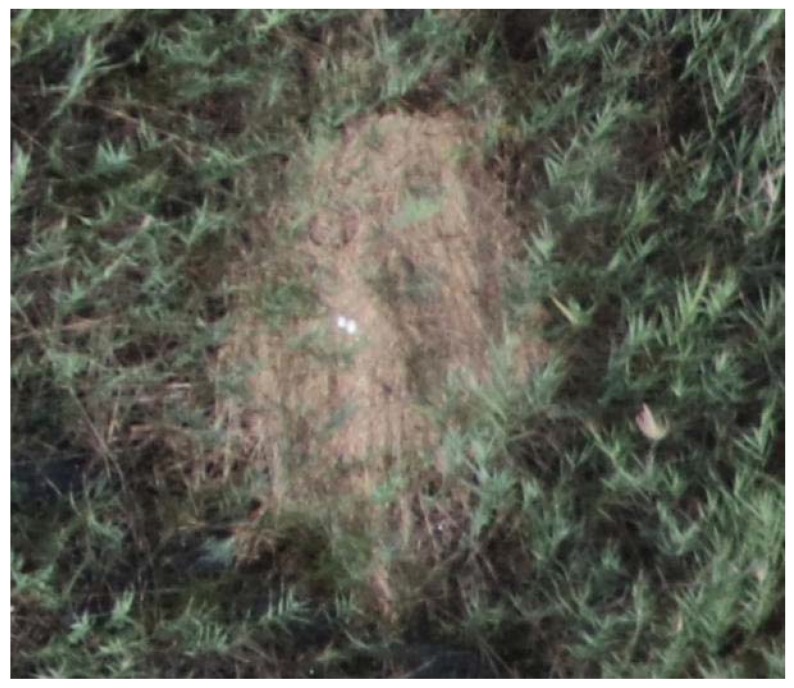
Nest with eggs. Isolated nest with eggs—details of an orthophoto with 0.7 cm spatial resolution.

**Figure 10:**
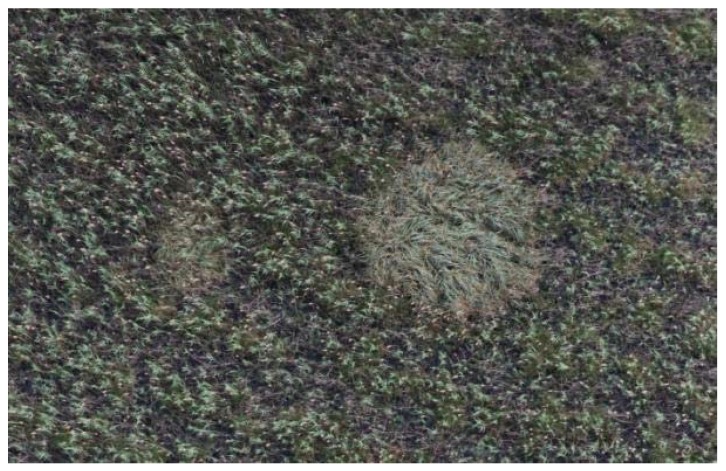
Nest-like vegetation structures. The analysis of small or medium resolution images was made more difficult by the appearance of nest-like vegetation structures, which could be easily recognized in images made with 0.7 cm spatial resolution.

The eggs that were not in the coverage could be counted in those orthophotos whose resolution was less than one cm detailed (Figure 9). In the orthophotos with 0.7 cm spatial resolution, it was not difficult to separate the vegetation spots (Figure 10) and the fishing spots from the nests.

If we considered the 0.7 cm GSD orthophoto made simultaneously with the oblique camera technique to be a reliable source of data, only 11.7% of the individuals in the 7–10 cm GSD orthophoto were identifiable, while the 1.5 cm GSD orthophoto detected only 21% of the individuals.

In the 7–10 cm GSD orthophoto, we could identify 80% of the nests, which improved to 81% by increasing the spatial resolution to 1.5 cm.

**Figure 11:**
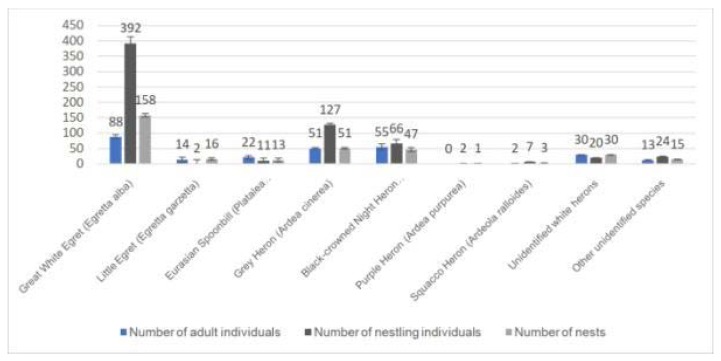
Results with additional oblique photogrammetry at Lake Kolon sample area. The distribution of individuals and nests in the observed area identified in the orthophotos made with the oblique camera technique with 0.7 cm spatial resolution.

If we did not consider the Pygmy Cormorants and concentrate exclusively on herons, the 7–10 cm spatial resolution surveys detected 20.9%, while the 1.5 cm spatial resolution surveys detected 37.5% of those individuals that were identified with 0.7 cm spatial resolution ortho-oblique technology (Table 5). The 0.7 cm spatial resolution survey detected 99.6% and correctly classified into species 93% of the heron individuals with ortho-oblique technology. In the case of nests, the difference was not so significant. The 2018 survey results of Lake Kolon’s heron colonies are shown in Figure 11.

**Figure 12:**
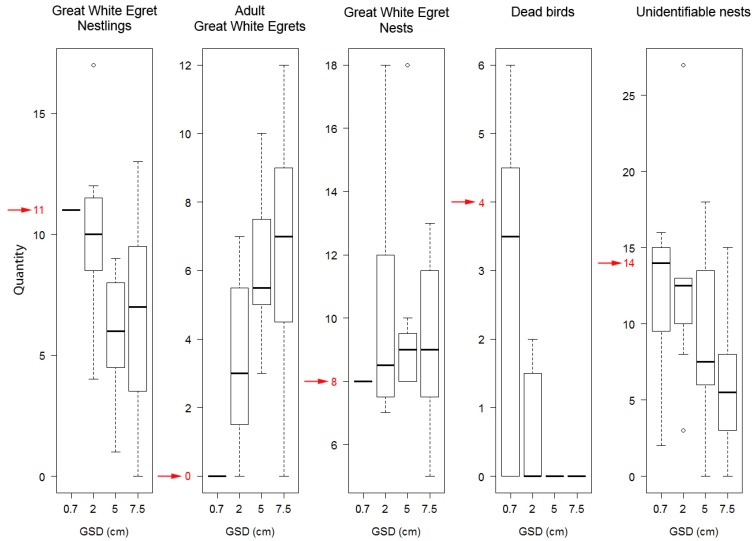
Deviation of the analysis of 8 interpreters, depending on the spatial resolution. Orthophotos with 0.7 cm spatial resolution were already sufficiently reliable to analyze the number of herons and heron nestlings. The red arrow indicates the values experienced in field inspection.

Since the accuracy of the analysis of the highest spatial resolution orthophoto and oblique imagery was not verifiable, a sub-area analysis was repeated with field validation. In June 2019, hail caused serious damage to the colonies of Lake Kolon. Since we had to check the colony after the hail, we could test the reliability of the high-resolution aerial survey method in one of the smaller colonies with the help of field inspection, and it was possible to prove the spatial resolution dependence of the accuracy. At that time, no adult herons were in the vicinity of the nests because of the field inspection, and the adult birds left the colony. The overflights were also carried out during the field survey for comparison. In the case of that separate Great White Egret breeding colony, the interpretation was performed with eight different analysts from small spatial resolution to large. Examining the subjective effect of the evaluators, it was found that orthophotos with 0.7 cm spatial resolution were already sufficiently reliable to analyze the number of colonies (Figure 12).

The experiments pointed out that a reliable counting procedure in the case of heron colonies could consist of the elaboration of an orthophoto map with 0.7 cm spatial resolution, with 90% overlap in line and at least 40% overlap between flight lines, made from high altitudes (450–800 m) and followed by a GIS (geographic information system)-based evaluation of the colony.

### 3.1. Establishing the Criteria of the Survey

The remote sensing platform can be a large-sized fixed-wing UAV or a pilot-driven aircraft. The most important requirements for the flying platform are to fly steadily and minimize roll (max. 1–2 degrees). It should carry securely a 6–25 kg (depends on the model, with or without oblique cameras) aerial camera system. Depending on the size of the breeding site, the flying groundspeed needs to be at least 160–270 km/h to prevent birds from significantly shifting until the aircraft returns to the next flight line. Ground speed is very important because more than 50 breeding sites, each are more hectares, need to be surveyed in a given time period per day. Depending on the species and habitat, it must fly at least 500–800 m above the ground so that the frequent surveys will not disturb the birds. It should be able to work in special weather conditions, as we need to survey a large number of colonies in a specific time period. As a result, those lenses that can be used in non-ideal lighting conditions, wide-aperture telephoto lens, and lenses that are able to make sharp and detailed images even from large distances from the object make up 80% of the equipment weight. The most important requirements for the camera system are its fast readout and recording speed and high sensitivity sensor with low noise levels, which provides dynamic and well-exposed images with short shutter speed. Care should be taken to ensure the proper plane alignment of the sensor and the quality of the minimally distorted optical system. It is very important to use direct orientation because there is no possibility for the measurement of field ground control points near the breeding sites. The direct georeferencing with GNSS (global navigation satellite system) post-processing enables the production of orthophotos accurate to the cm, while without correction, the extent of the accuracy is half a meter without carrying out field measurements.

For the production of orthophotos, any modern photogrammetric software that is operating on visual SfM (Visual Structure from Motion System) principle is recommended. The visual interpretation, which is the geoinformatic evaluation of the orthophotos, can be performed with any GIS software, but we primarily recommend software that enables the changing of the histograms of the images during the evaluation. This offers a great deal of support because the dynamics of the photographs allow us to bring hidden information, for example, by darkening the image to sharpen the yellow spot on the back of a Eurasian Spoonbill.

Interspect IS 5 digital aerial cameras were used with 300 mm and 500 mm focal length lenses, but experiments were conducted with a number of small and medium format cameras that could continuously shoot at exposures intervals of less than half a second (exposition interval). Leading camera manufacturers (Nikon, Canon, Sony, etc.) offer such camera bodies available in any country, as well as high-quality lenses (e.g., AF-S Nikkor 600 mm f/4E FL ED, AF-S Nikkor 500 mm f/4E FL ED VR, AF-S Nikkor 400 mm f/2.8E FL ED VR, Nikon AF-S 300 mm f/4E PF ED, Canon EF 600 mm f/4L IS II USM, Canon EF 500 mm f/4L IS II USM, EF 400 mm f/2.8L IS II USM, Canon EF 300 mm f/2.8L IS II USM, Sony 500 mm f/4G, Sony SEL FE 400 mm f/2.8, Sony 300 mm f/2.8G, etc.).In our experience, a camera body with a higher resolution sensor (40+ MP)—a smaller focal length (300–400 mm) lens is preferable because longer focal length lenses with a few degree fields of view make the surveys extremely sensitive to aircraft roll. Images were processed with an Agisoft Photoscan photogrammetric software and analyzed in Quantum GIS software.

### 3.2. Effect of the Application of Oblique Photogrammetry on the Accuracy of Interpretation

**Figure 13:**
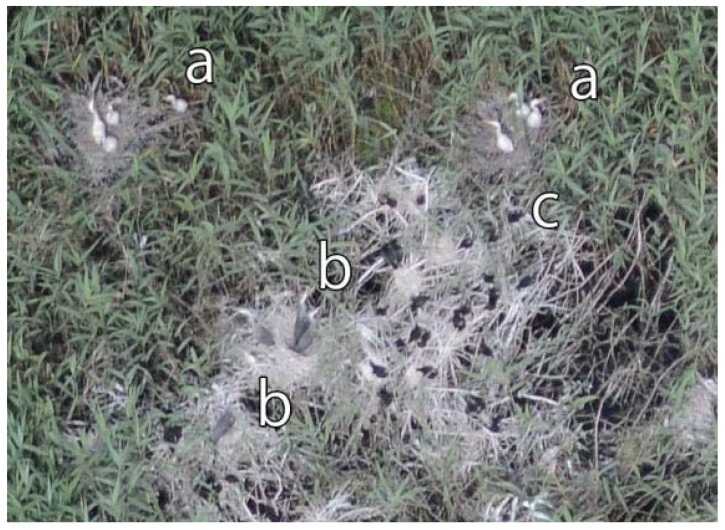
Detail of an oblique image. An oblique camera axis image with (**a**) Great White Egret nestlings, (**b**) Grey Heron nestlings, and (**c**) Pygmy Cormorant nestlings.

If oblique camera axis images were made from two directions, the identification of individuals increased by 4.55%. This was primarily due to the fact that the identification of 14% of those individuals that were classified as “other species” (that cannot be determined exactly) during the analysis of a simple orthophoto was becoming feasible by the simultaneous analysis of orthophotos and oblique camera axis images. Through the use of this technique, the isolation of Great White Egrets and Eurasian Spoonbills was 28% more reliable. The definition of White Herons improved by 31%, but the identification of Grey Herons was only more accurate by 0.5%. This survey that was made using the oblique photogrammetric technique was an essential element in the counting of individuals (Figure 13).

In case of the monitoring the nests of Lake Kolon, the simultaneous application of images with a vertical and bi-directional oblique axis resulted in an improvement of 8.41% in reliability.

The greatest improvement (15%) in the detection of Eurasian Spoonbill nests was due to the simultaneous application of oblique and vertical axis orthophoto technology. The total number of White Heron nests could be determined 21% more precisely with the mentioned method. The identification of Great White Egret nests improved by 6%. It is worth mentioning that the Purple Heron nests could only be detected through the use of this complex method; with a simple orthophoto-based analysis, we could not distinguish Purple Heron individuals from Grey Herons.

In addition to a one-time investment, it does not create any significant increase in costs to make captures with an oblique camera axis while taking high-resolution digital photographs with a vertical camera axis. Considering the minimum increase in costs and the impact of the method on the reliability of the survey, we recommend applying this technology for the monitoring of heron colonies.

### 3.3. Limitations and Possibilities of Use

The presented method allows non-disturbing bird colony-mapping in case of nesting areas where the canopy level does not cover the nests. An inspection of the number of individuals, repeated three times per year, and an annual inspection of the number of nests could play an important indicator role in the area of nature and landscape protection. The method could also contribute to the protection of the adjacent areas and be important because the number of populations does not only depend on the quality of the direct breeding sites.

## 4. Conclusions and Discussions

Previously, the creation of orthophotos with sufficient overlap and ultra-detailed spatial resolution was not accessible at high flight speed and at high ground speed. In the present work, we developed a practical environmental application of the method that we introduced in 2014 [8]. With our method, it was feasible to survey larger areas, and 0.7 cm spatial resolution orthophotos were provided at higher groundspeed. As a result, our method could be used even if the exact location of the colonies is unknown. With the use of the present technique, high-resolution imagery is e feasible even under a flight altitude of 100 m, as in the case of several former researches (for example [4,25]). In the present study, we did not apply computer-automated image analysis (like [11]), because our aim was to develop a method that is able to make high quality and large spatial resolution images from high altitudes.

The simultaneous use of the subcentimeter spatial resolution orthophoto mapping and oblique mapping method followed by a GIS-based evaluation improved the precision of individual counting by at least 60%, made the analysis of the nest numbers more accurate, and even made it feasible to classify the nests. Compared to those field and aerial surveys that were made in recent years, the survey reliability of Lake Kolon’s heron colonies improved considerably, and the survey provided far more parameters for Nature Conservation Information Systems. The non-disturbing, high-speed process from an altitude of 450–800 m was not only more reliable than previous methods but provided more detailed information without disturbing the animals.

We recommend three aerial surveys per year in the case of important breeding sites, which are remarkable from the perspective of nature conservation and which are good environmental indicators in the period from May to June because with disturbance-free methods, it is possible to track the status of the populations without risking the living conditions of birds and by minimizing the amount of stress. It would be worthwhile to investigate the possibilities of evaluating orthophotos based on an automated or semi-automated image analysis algorithm in independent research. It is also worth adding to the procedure with thermal imagers, although due to the high flight altitude, we do not expect such good results as we see in the thermal counting works [26,27].

An algorithm, which allows distinct small signals and features, has great potential in accelerating evaluation, irrespective of the position, size, and rotation of the object, as well as robust affinity transformations (including changes in scale, rotation, size, and position) and lighting conditions. There are methods for Spatio-temporal analysis of image series for the exploration of relationships [28], so automatic analysis of the orthophoto series from flight sequence to flight sequence can provide better results than analysis of orthophoto mosaics.

During rapid recording, 6 to 10 consecutive shots also record the movement and change of position of the individual birds as the aircraft passes. This allows the use of action classification algorithms too.

## Figures and Tables

**Table 1:** Spatial resolution (cm/pixel) as a function of relative flight altitude and focal length. Hrel: altitude above ground.

	Focal Length						
Hrel (m)	50 mm	100 mm	200 mm	300 mm	400 mm	500 mm	600 mm
1200 m	11.71	5.85	2.93	1.95	1.46	1.17	0.98
1100 m	10.73	5.37	2.68	1.79	1.34	1.07	0.89
1000 m	9.76	4.88	2.44	1.63	1.22	0.98	0.81
900 m	8.78	4.39	2.19	1.46	1.10	0.88	0.73
800 m	7.80	3.90	1.95	1.30	0.98	0.78	0.65
700 m	6.83	3.41	1.71	1.14	0.85	0.68	0.57
600 m	5.85	2.93	1.46	0.98	0.73	0.59	0.49
500 m	4.88	2.44	1.22	0.81	0.61	0.49	0.41
400 m	3.90	1.95	0.98	0.65	0.49	0.39	0.33

**Table 2:** Longest shutter speed without motion blur (1/x s) as a function of relative flight altitude and focal length at 160 km/h ground speed. Hrel: altitude above ground.

	Focal Length						
Hrel (m)	50 mm	100 mm	200 mm	300 mm	400 mm	500 mm	600 mm
1200 m	379.7	759.3	1518.6	2277.9	3037.2	3796.6	4555.9
1100 m	414.2	828.3	1656.7	2485.0	3313.4	4141.7	4970.0
1000 m	455.6	911.2	1822.3	2733.5	3644.7	4555.9	5467.0
900 m	506.2	1012.4	2024.8	3037.2	4049.7	5062.1	6074.5
800 m	569.5	1139.0	2277.9	3416.9	4555.9	5694.8	6833.8
700 m	650.8	1301.7	2603.4	3905.0	5206.7	6508.4	7810.1
600 m	759.3	1518.6	3037.2	4555.9	6074.5	7593.1	9111.7
500 m	911.2	1822.3	3644.7	5467.0	7289.4	9111.7	10934.1
400 m	1139.0	2277.9	4555.9	6833.8	9111.7	11389.7	13667.6

**Table 3:** Swath width (m) as a function of relative flight altitude and lens focal length. Hrel: altitude above ground.

	Focal Length						
Hrel (m)	50 mm	100 mm	200 mm	300 mm	400 mm	500 mm	600 mm
1200 m	861.6	430.8	215.4	143.6	107.7	86.2	71.8
1100 m	789.8	394.9	197.5	131.6	98.7	79.0	65.8
1000 m	718.0	359.0	179.5	119.7	89.8	71.8	59.8
900 m	646.2	323.1	161.6	107.7	80.8	64.6	53.9
800 m	574.4	287.2	143.6	95.7	71.8	57.4	47.9
700 m	502.6	251.3	125.7	83.8	62.8	50.3	41.9
600 m	430.8	215.4	107.7	71.8	53.9	43.1	35.9
500 m	359.0	179.5	89.8	59.8	44.9	35.9	29.9
400 m	287.2	143.6	71.8	47.9	35.9	28.7	23.9

**Table 4:** Geometric accuracy tested on grassland and urban areas. ICPs: number of independent checkpoints, GCPs: number of ground control points, RMSE: planimetric root mean square error, HCEmax: maximum horizontal circular error (distance between ICP and its image sign).

Surveyed Sample Area	Number of Images	Points Measured in the Field		Spatial Resolution	Planimetric Accuracy	
		ICP	GCP		RMSE	HCEmax
60,000 m^2^	246	60	0	0.7 cm	11.7 cm	17.4 cm
60,000 m^2^	246	60	4	0.7 cm	5.11 cm	8.7 cm
60,000 m^2^	42	60	0	2 cm	17.25 cm	31.4 cm
60,000 m^2^	42	60	4	2 cm	5.42 cm	13.4 cm
18,000,000 m^2^	673	15	0	7.5 cm	20.7 cm	37 cm
5,525,000 m^2^	242	17	14	7.5 cm	11.7 cm	30 cm

**Table 5:** Comparative results. The number of detected individuals and nests in the observed area based on orthophotos with different spatial resolutions.

Spatial resolution	Adult Individuals	Nestling Individuals	Number of Nests
7–10 cm	124	70	336
1.5 cm	156	192	336
0.7 cm	274	652	336
0.7 cm orthophoto made with the oblique technique	276	654	337
